# Unlocking Resveratrol's Potential: Targeting Ferroptosis in Atherosclerosis Through MAPK1

**DOI:** 10.1002/fsn3.70466

**Published:** 2025-07-21

**Authors:** Yao Zhang, Jun Cheng, Wu Jian

**Affiliations:** ^1^ Department of Inspection Changde Hospital, Xiangya School of Medicine, Central South University (The first people’s hospital of Changde city) Changde Hunan China

**Keywords:** atherosclerosis, ferroptosis, machine learning, MAPK1, molecular docking, resveratrol

## Abstract

Atherosclerosis (AS) is a chronic inflammatory metabolic disorder and a leading cause of cardiovascular diseases. Resveratrol (RSV), a natural polyphenolic phytoestrogen, exhibits anti‐atherosclerotic effects by modulating oxidative stress and ferroptosis, yet its key therapeutic targets remain unclear. Using network pharmacology, bioinformatics, machine learning, and molecular docking, we identified core targets and mechanisms of RSV in ferroptosis and anti‐atherosclerosis. Experimental validation was performed using ApoE^−/−^ mouse fed a high‐fat diet (HFD) for 12 weeks to establish AS model. We assessed aortic and aortic root plaque formation, serum oxidative stress, and iron levels. By mining online databases, we identified 31 shared targets at the intersection of RSV‐AS‐Ferroptosis. A PPI network was generated using STRING, and GeneMANIA, GO and KEGG analyses revealed key biological processes and pathways (such as oxidative stress). Employing eight machine learning algorithms, we pinpointed six key targets: MAPK1, IL1B, RELA, HIF1A, SRC, and PTEN. Differential gene docking and molecular docking analyses showed that MAPK1 (−8.8 kcal/mol binding energy) had relatively good affinity. In vivo, RSV treatment reduced aortic lipid plaques, reduced serum GSSG/GSH, SOD, MDA, and iron levels, and significantly downregulated MAPK1 expression in the aortic root. RSV could modulate the ferroptosis pathway through targeting the MAPK1 gene, providing a new theoretical framework for AS prevention and treatment.

## Graphical abstract

Using network pharmacology, bioinformatics, machine learning, and molecular docking, we identified mechanisms and core six key targets (MAPK1, IL1B, RELA, HIF1A, SRC, and PTEN) in RSV‐AS‐Ferroptosis. Experimental validation was performed using ApoE^−/−^ mouse fed a high‐fat diet (HFD) for 12 weeks to establish AS model. RSV treatment reduced aortic lipid plaques, reduced serum GSH, SOD, MDA and iron levels, and significantly downregulated MAPK1 expression in aortic root, which providing a new theoretical framework for AS prevention and treatment.

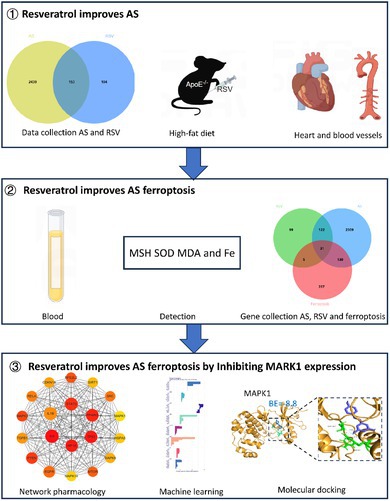

## 1 Introduction

Cardiovascular disease (CVD) is a leading cause of global mortality, primarily driven by atherosclerosis (AS), a progressive metabolic disorder disease (Pervaiz et al. 2023; Pan, Ho, et al. 2024). The hallmark of AS—arterial plaque formation—is closely linked to pathophysiological processes such as chronic inflammation and oxidative stress, which play pivotal roles in triggering cardiovascular events (Darwitan et al. 2020; Steenman et al. 2018). While statins and other lipid‐lowering agents remain first‐line treatments for AS, their long‐term use is often limited by drug resistance and adverse effects, potentially undermining therapeutic outcomes and even increasing patient mortality (Nelson et al. 2022). Given these challenges, advancing the understanding of AS pathogenesis, developing novel therapeutics, and optimizing prevention and treatment strategies have emerged as critical priorities in cardiovascular research.

Active compounds derived from traditional Chinese medicine (TCM) show significant potential in preventing and treating atherosclerosis (AS), owing to their high efficacy and low toxicity. Among these, resveratrol (3,4,5‐trihydroxystilbene, RSV), a lipid‐soluble non‐flavonoid polyphenol, exhibits a unique stilbene structure resembling the synthetic estrogen diethylstilbestrol. It exists in three isomeric forms—*cis*, *trans*, and piceid—and is abundant in grapes, berries, peanuts, and various medicinal plants. Its association with the “French paradox” highlights its role in cardiovascular protection, as it helps explain the low incidence of CVD despite high dietary cholesterol and saturated fat intake (Dikmetas et al. 2024). Research has demonstrated that resveratrol exerts multiple cardioprotective effects, including antioxidant, anti‐inflammatory, and anti‐atherosclerotic properties (Fan et al. 2022). Relevant studies have shown that resveratrol reduces apoptosis, lipid accumulation, and adhesion, thereby reversing cell dysfunction induced by ox‐LDL (Yu and Fang 2022). Resveratrol has been shown to attenuate angiotensin II (Ang‐II)‐induced atherosclerotic plaque formation in ApoE^−/−^ mice; furthermore, it suppresses monocyte‐to‐macrophage differentiation and mitigates inflammation by restoring cellular glutathione (GSH) levels, thereby offering protective effects against atherosclerosis (Jing et al. 2023; Vasamsetti et al. 2016).

Ferroptosis is a distinct form of programmed cell death driven primarily by iron‐dependent phospholipid peroxidation and tightly regulated by lipid metabolism, the antioxidant system, iron homeostasis, and multiple signaling pathways. Emerging evidence highlights its critical role in the pathogenesis of AS, with molecular mechanisms closely linked to the expression of MAPKs family genes (Huang et al. 2021). Specifically, ox‐LDL triggers ferroptosis by suppressing glutathione peroxidase 4 (GPX4) activity, while ferroptosis inhibitors can mitigate this process, ameliorating hyperlipidemia and slowing AS progression (Luo et al. 2024).

These findings suggest that targeted inhibition of ferroptosis may represent a promising therapeutic strategy against atherosclerotic plaque formation. Notably, RSV exhibits potential in modulating ferroptosis. For instance, RSV has been shown to alleviate 5‐fluorouracil (5‐Fu)‐induced cardiotoxicity by suppressing the GPX4‐dependent ferroptotic pathway (Li et al. 2023). Additionally, RSV inhibits ferroptosis via regulation of the microtubule affinity regulating kinase (MAPK) signaling cascade, offering protection against adriamycin‐induced cardiotoxicity (Chen et al. 2024). However, the precise molecular mechanisms and targets through which RSV regulates ferroptosis in AS remain unclear.

**FIGURE 1:**
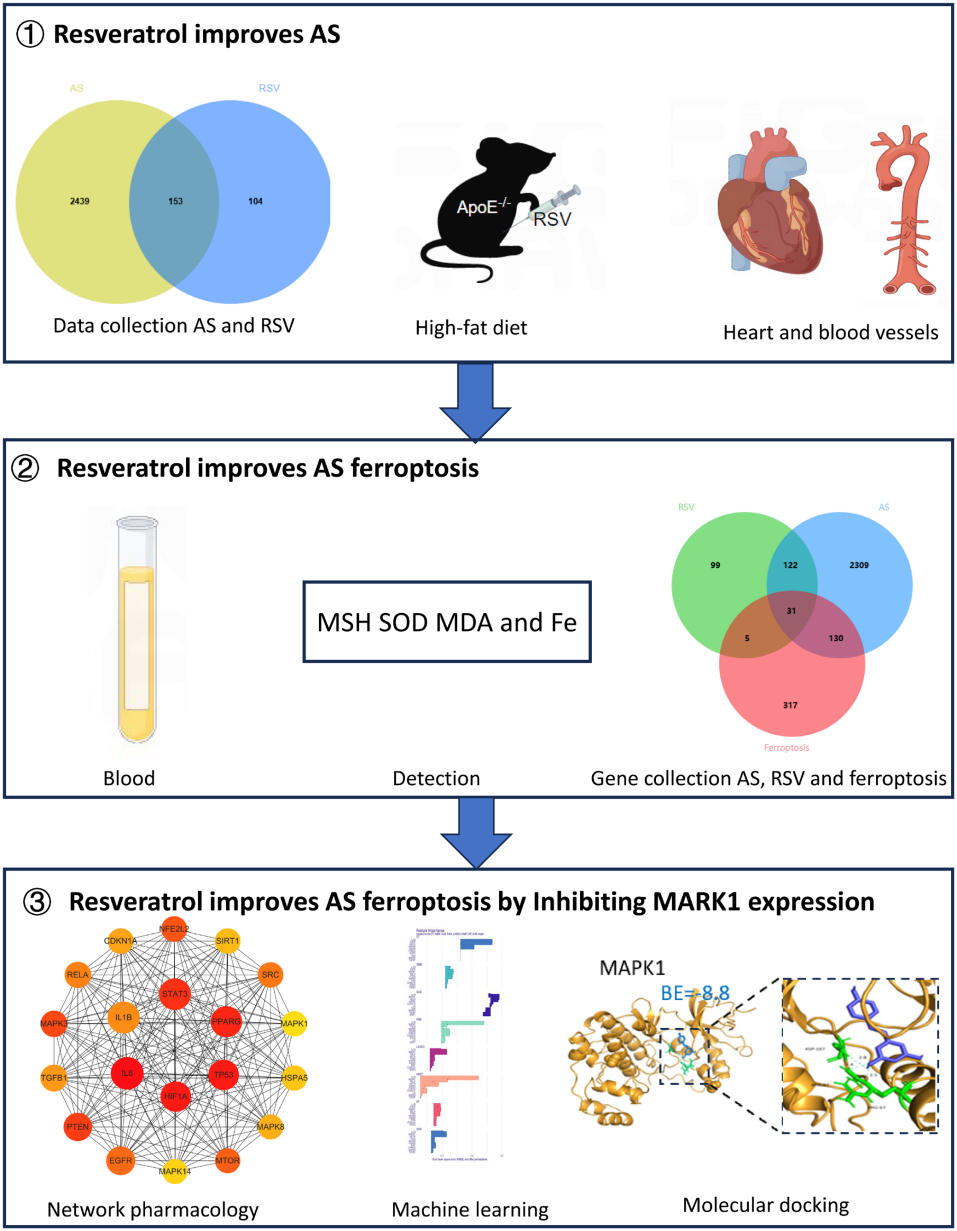
Graphical abstract.

To investigate the role of RSV‐regulated ferroptosis in AS, this study employed a multidisciplinary approach combining network pharmacology, bioinformatics analysis, machine learning‐based prediction, and molecular docking techniques, along with experimental validation. This comprehensive strategy was designed to elucidate the molecular mechanisms underlying RSV‐mediated ferroptosis in AS, thereby providing a crucial biological foundation for the development of novel AS therapeutics (Figure 1).

## 2 Materials and Methods

### 2.1 RSV‐Related Targets Collection

To identify potential targets of resveratrol (RSV), we conducted a comprehensive search across multiple drug‐related databases using “Resveratrol” as the keyword. The databases included: TCMSP (Traditional Chinese Medicine Systems Pharmacology Database, http://www.tcmsp‐e.com/): identified 151 RSV‐related targets. ETCM (Encyclopedia of Traditional Chinese Medicine, http://www.tcmip.cn/ETCM/): retrieved 25 RSV‐associated targets. PharmMapper (https://lilab‐ecust.cn/pharmmapper/): selected the top 50 targets (Norm Fit > 0.6). SwissTargetPrediction (http://www.swisstargetprediction.ch/): obtained 69 targets (probability > 0). After removing duplicates, we retained 257 unique RSV‐related targets for further analysis.

### 2.2 AS Potential Targets Collection

Using online Mendelian inheritance human disease related database (OMIM, https://www.ncbi.nlm.nih.gov/omim/) and GeneCards (https://www.genecards..org/) for Atherosclerosis related gene. In addition, AS data set GSE23746, GSE100927, GSE125771 from the national center for biotechnology information (NCBI) gene expression database (GEO, http://www.ncbi.nlm.nih.gov/geo) to download, in order to determine the AS related targets, OMIM and GeneCards databases were used to obtain AS data targets, and the Relevance score was taken as the continuous median to obtain the target. AS samples were downloaded from the GEO dataset for DEG analysis. The surrogate variable analysis (sva) algorithm was used to directly remove batch effects from datasets GSE100927, GSE125771 and to identify DEGs. Wgcna‐related genes and DEGs identified by Limma cross‐set genes were performed on GEO‐AS samples.

### 2.3 FRGs Collection

We enabled FerrDb, a database of ferroptosis related markers, regulators and curation and identification, to collect ferroptosis‐related genes (FRGs).

### 2.4 DEGs Analysis

Differentially expressed gene analysis was performed using the “Limma” package of R software (version: 3.40.2) (https://www.home‐for‐researchers.com/#/). Genes meeting the criteria of |log (fold change, FC)| > 1.3 and *p* < 0.05 were defined as differentially expressed genes (DEGs). The datasets used in this study are sourced from the GEO database (https://www.ncbi.nlm.nih.gov/geo/), with the downloaded data in MINiML format. The detailed processing procedure can be found in the method description on the dataset selection page.

### 2.5 WGCNA Analysis

Bioinformatics methods were used to describe gene correlation patterns among samples. The WGCNA software package was used for weighted gene co‐expression network analysis of the GEO dataset.

### 2.6 RSV‐AS‐Ferroptosis Potential Targets Collection

Through the Swiss target Prediction (http://SwissTargetPrediction), ETCM (http://www.tcmip.cn/ETCM/index.php/Home/), TCMSP (http://www.tcmsp‐e.com/index.php) and Pharmapper (https://lilab‐ecust.cn/pharmmapper) database for drug target identification and generated 257 potential AS‐related targets after deduplication. The conversion of protein names to gene symbols is provided by the UniProt database. Venn diagram analysis identified 31 unique targets shared by RSV and AS as well as ferroptosis, setting the stage for further analysis.

### 2.7 Functional Analyses

We used Gene ontology (GO), Kyoto encyclopedia of genes and genomes (KEGG), and GeneMANIA to functionally analyze the comprehensive network of target genes and signaling pathways. The R package was used for GO enrichment and KEGG functional annotation studies and visualization studies, and the “ClusterProfiler” R package was frequently used for functional annotation studies. The “ggplot” R package was used for visual analysis. We also used GeneMANIA (https://genemania.org/) for functional analyses.

### 2.8 Construct PPI Network

The Search Tool for The Retrieval of Interacting Genes (STRING, http://string‐db.org) web tool was used to determine protein associations. Here, we import FRGs into STRING and set species to *Homo sapiens* to obtain protein–protein interaction PPI networks. Subsequently, it was further visualized and analyzed in Cytoscape. In addition, RSV‐AS‐FRGs interaction networks were generated using Cytoscape. The top 20 key targets were identified as hub targets using the CytoHubba plugin, and the degree algorithm was used to evaluate the biological significance. This approach ensures a robust and high‐confidence network analysis to identify key therapeutic targets for AS.

### 2.9 Machine Learning

To identify candidate diagnostic biomarkers for AS, computational methods that automatically improve and optimize model performance on training data of DT, GBM, GLM, KNN, LASSO, NNET, RF, and SVM were combined to screen potential biomarkers. We used the top 20 genes obtained by the Cytoscape‐based MCC algorithm in the PPI network as input to perform expression profiling in the GSE100927 and GSE125771 datasets with eight machine learning algorithms. We used the “glmnet” package in R software for LASSO analysis, the “randomForest” package for RF analysis, the “e1071” package for SVM recursive feature elimination (SVM‐RFE) analysis, and the “caret” package for NNET analysis. Finally, the overlapping genes identified by the first four machine learning algorithms were defined as biomarkers for AS.

### 2.10 Molecular Docking

Molecular docking and intermolecular interaction analysis methods are essential to assess the binding capacity and intermolecular interaction between proteins and ligands. From the PubChem database (https://pubchem.ncbi.nlm.nih.gov/) to download RSV three‐dimensional structure, MAPK1 (−8.8 kcal/mol), IL1B (−6.3 kcal/mol), RELA (−6.6 kcal/mol), HIF1A (−6.3 kcal/mol), SRC (−7.1 kcal/mol), and PTEN (−7.1 kcal/mol) were obtained from the PDB database (https://www.rcsb.org/) (kcal/mol). The docking was performed using Autodock Vina software and visualized by PyMOL software. In addition, Autodock Vina molecular docking results were imported into LigPlot+ software to explore the intermolecular interactions between the ligand (RSV) and the proteins MARK1, IL1B, RELA, HIF1A, SRC, and PTEN.

### 2.11 Materials

Resveratrol (MedChemExpress, SRT501) was dissolved in 1% DMSO for in vivo experiments and in EtOH, 10%–90% (20% SBE‐β‐CD in saline) for in vitro experiments; primary antibodies against MAPK1 (F1152) from Selleck Biotechnology (Houston, Texas, USA), Polymer HRP (Rabbit) IHC Kit (DAB)‐P (RS0063) from Immunoway Biotechnology (Tennyson Pkwy Ste., Plano, TX, USA); OCT embedding agent, hematoxylin, and eosin were purchased from Servicebio (Optics Valley Biology City, Wuhan, Hubei, China), and serum iron kit (24101407) was purchased from Beijing Lidman Biochemical Co. Ltd. (Beijing, China). SOD (S0101S), MDA (S0131S), and GSH/GSSG (S0053) kits were purchased from Biyuntian Company (Songjiang District, Shanghai, China).

### 2.12 Model Establishment

Twelve healthy 8‐week‐old ApoE^−/−^ mice (purchased from Nanjing Jici Pharmaceutical Kang Co. Ltd., body weight (20 ± 5)g) were selected and fed with a HFD diet with free water and food. Before the formal experiment, the rats were fed in a conventional environment for a week (ventilation, temperature 18°C–25°C, humidity 40%, light and dark time 12 h each). They were divided into a high‐fat control group (HFD) (*n* = 6) and a high‐fat plus RSV treatment group (HFD + RSV) (*n* = 6). 8‐week‐old ApoE^−/−^ mice were induced by high‐fat diet for 12 weeks to establish atherosclerosis animal models. From the 8th week, 50 mg/kg RSV or saline was injected daily for 4 weeks. Experiments were performed in accordance with relevant institutional and national guidelines for the care and use of laboratory animals. All animal experiments were approved by the Experimental Animal Ethics Committee, University of South China (Ethics Approval Number: USC2024XS286).

### 2.13 Hematoxylin–Eosin Staining

After ApoE^−/−^ mice were sacrificed by anesthesia with an overdose of sodium pentobarbital, hearts were rapidly removed and labeled and placed in 30% sucrose for 36 h of dehydration. After dehydration, the slices were cut in the middle, embedded in OCT, sectioned in a frozen section mechanism with a thickness of 6 μm, mounted on a special glass slide, and stained with HE after gradient dehydration: Absolute ethanol →95% ethanol →85% ethanol →70% ethanol → hematoxylin 5–30 s → pure water to develop slides → 0.5% hydrochloric acid ethanol differentiation 1–10 s → tap water to turn blue → pure water to develop slides →70% ethanol → eosin 2–20 min → pure water to develop slides →70% ethanol →95% ethanol → neutral gum sealing slides → Immediately film.

### 2.14 Oil Red O Staining

Oil red O was dissolved in 60% isopropanol, filtered, and used. Samples of full‐length aortic vessels were taken, the entire aorta was dissected with microscissors, fixed with a fine needle, immersed in 60% isopropanol for 5–10 s, incubated with oil red O solution for 3–5 min, differentiated by adding 60% isopropanol solution for 10–15 s, washed three times with distilled water, lipids were red, and photographed.

### 2.15 Serum Fe, SOD, MDA, and GSSG/GSH Levels

Blood was collected from the eye margin, and the supernatant was centrifuged for determination of serum Fe, SOD, MDA, and GSSG/GSH levels according to the requirements of the kits.

### 2.16 Tissue Immunostaining

For chromogen staining, sections were fixed in 10% formalin for 10 min, then Microwave repair for 2 min, and repeated three times. Slides were blocked with 3% hydrogen peroxide for 10 min and in 5% bovine serum albumin for 1 h. Primary antibody incubations were carried out overnight at 4°C (MAPK1:1:200). Horseradish peroxidase‐conjugated secondary antibody (1:500) and DAB were used for detection. Then, the experiments were completed according to the requirements of the Polymer HRP (Rabbit) IHC Kit (DAB)‐P kit. Image J (NIH; Bethesda, MD, USA) determined the staining‐positive area.

### 2.17 Statistical Analysis

Data are reported as mean ± SEM. GraphPad Prism 9.0 software (San Diego, CA, USA) was used for statistical analysis, and t test was used to compare the means of the two groups. A P value of less than 0.05 was considered statistically significant.

## 3 Results

### 3.1 Collection Targets of RSV and AS

**FIGURE 2:**
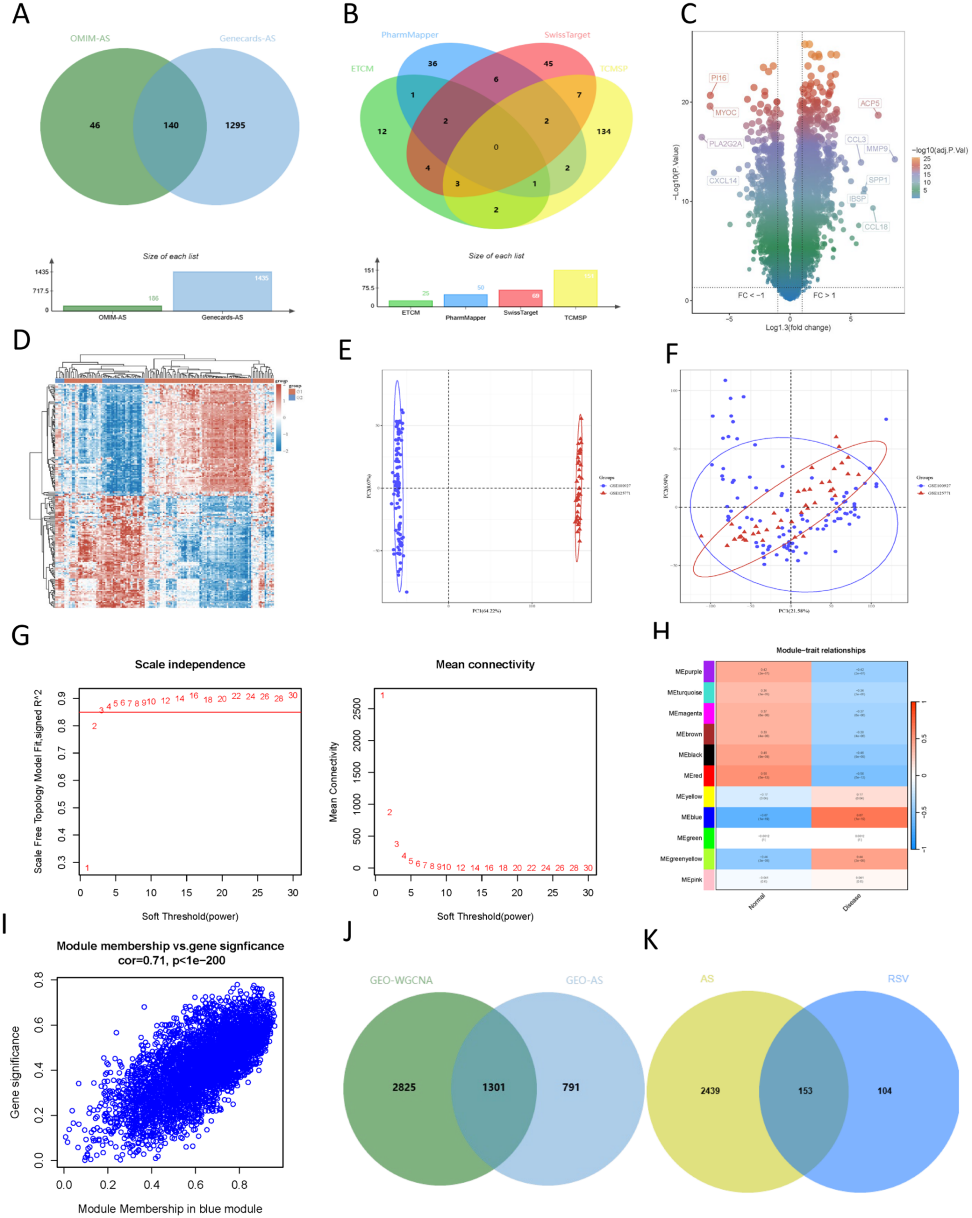
Predicted targets of RSV in relation to AS. (A) AS‐related targets in various disease databases (OMIM, and GeneCards). (B) Drug‐related databases (ETCM, TCMSP, PharmMapper and SwissTarget Prediction) explored the targets of RSV. Volcano (C) and heat map (D) of differentially expressed genes in GEO samples. The results of PCA analysis of the GEO dataset remove batch effects before (E) and after (F) rectification. WGCNA R package was used to construct a co‐expression network (G–I) in GEO samples. When the soft threshold was set to 3, the scale‐free topological fitting index (R2) was 0.85. A heat map of the relationship between the control and the AS module, with the modules represented in different colors. Red indicates a positive correlation, while blue indicates a negative correlation. (J) AS‐related genes obtained from the intersection of WGCNA and DEG in the GEO dataset (K) AS‐related genes obtained from the intersection of AS and RSV.

To identify genes associated with AS, we collected OMIM and GeneCards database associated genes, 186 AS associated genes in the OMIM. We obtained 5580 relevant targets in the GeneCards database and 1435 targets using the continuous median relevance score (Figure 2A). After deduplication, there were a total of 1481 target genes.

To obtain the targets of RSV, we searched drug‐related databases with the keyword “Resveratrol”. The results showed that there were 151 targets related to RSV in the TCMSP database, 25 in ETCM, the top 50 targets in PharmMapper (Norm Fit > 0.6), and 69 targets in SwissTargetPrediction (*p* > 0). After excluding duplications, there were 151 targets related to RSV in the TCMSP database, 25 targets related to RSV in the ETCM database, and 69 targets related to RSV in the SwissTargetPrediction database. A total of 257 targets were left (Figure 2B).

In order to improve the reliability of the results, we searched “Atherosclerosis” in the GEO database to obtain a total of 144 samples from GSE100927 and GSE125771 datasets for DEG analysis. We used the Limma package of R software (version: 3.40.2) to investigate the differential expression of mRNAs, and used the surrogate variable analysis (sva) algorithm to remove batch effects to generate principal component analysis (PCA) maps. A total of 2092 DEGs (of which 932 were down‐regulated and 1160 were up‐regulated) were identified (Figure 2C–F).

To identify the key modules associated with AS, we performed a scale‐free topological fit index of 0.85 for the co‐expression network when the soft threshold power was 3 for 144 samples analyzed by WGCNA (Figure 2G). In addition, 11 modules were identified, of which the blue module was highly correlated with AS (Figure 2H). Scatter plots show a significant relationship between the blue module and AS (cor = 0.71, *p* < 1e−200) (Figure 2I).

We obtained 1301 genes by crossing the WGCNA blue module related genes with the DEGs identified by Limma (Figure 2J), and then crossed the collection with targets obtained from OMIM, GeneCards databases and RSV‐related genes to obtain 153 RSV and AS core targets (Figure 2K).

### 3.2 Resveratrol has Anti‐Atherosclerotic Effects

**FIGURE 3:**
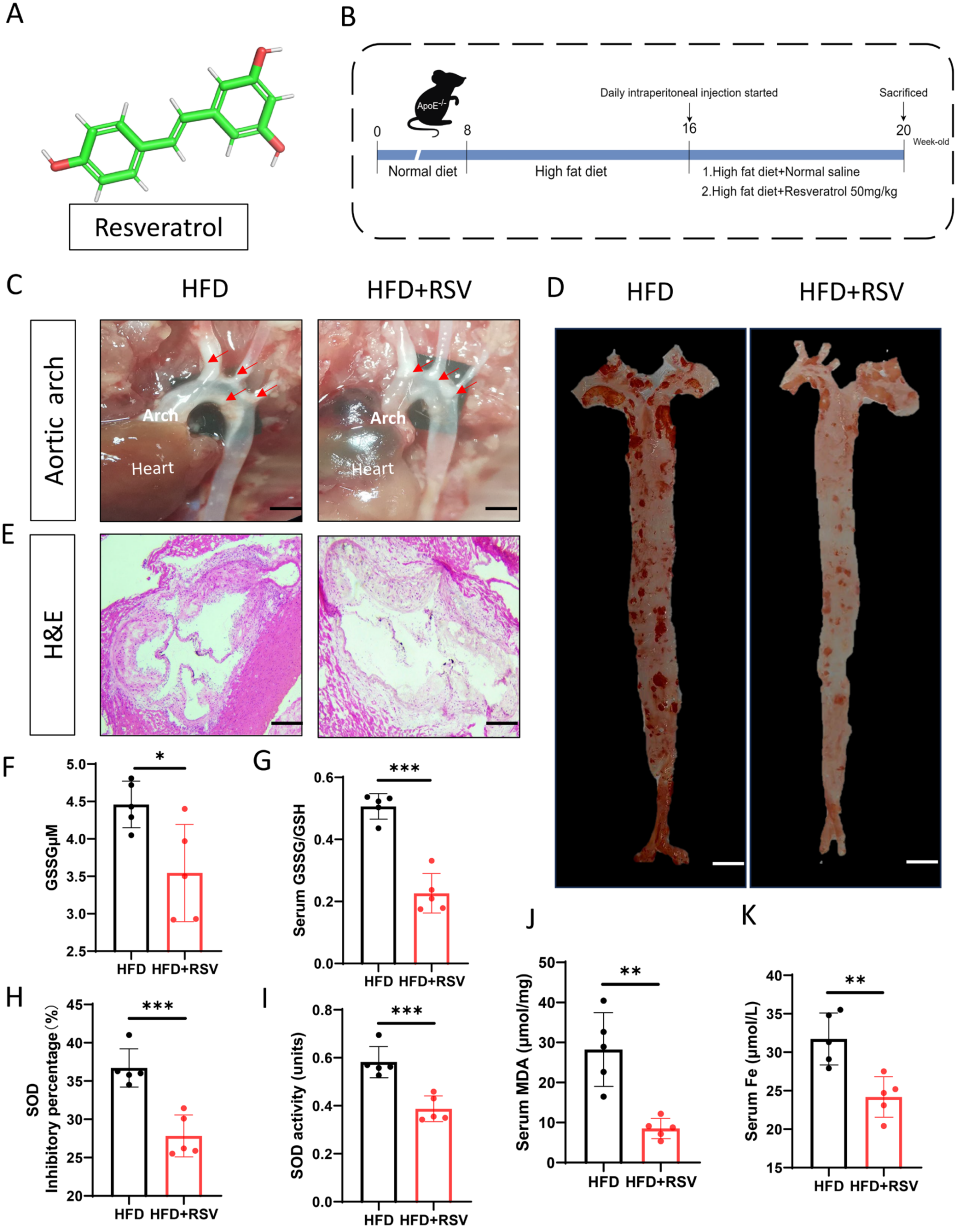
RSV improves atherosclerosis by reducing oxidative stress and iron metabolism. (A) Resveratrol molecular formula; (B) Experimental design; (C) Mouse aortic arch [scale bar = 1 mm]; (D) Oil red o staining the surface aorta of mice [scale bar = 2.5 mm]; (E) HE staining of aortic roots [scale bar = 250 μm]; Serum GSSG (F) and GSSG/GSH content (G) in mice; SOD inhibition rate (H) and enzyme activity (I); MDA content (J) and serum iron (K), *n* = 5. The data were expressed as mean ± SEM. *p < 0.05, **p < 0.01, ***p < 0.001.

To investigate the direct effects of RSV on AS, ApoE^−/−^ mice were used as an experimental model. In the present study, male ApoE^−/−^ mice were fed a HFD diet for 12 weeks to induce AS. Mice were assessed for the effect of RSV on the development and progression of AS by intraperitoneal injection of RSV (50 mg/kg) or saline during weeks 9–12 of the dietary regimen (Figure 3B). Our results showed that HFD feeding significantly promoted aortic plaque formation, whereas RSV treatment significantly attenuated aortic and aortic arch atherosclerotic lesions (Figure 3C–E). Results show that HFD feeding significantly promoted serum iron expression, activated SOD enzyme activity and its inhibition rate, promoted serum MDA expression, increased GSSG and GSSG/GSH, and serum iron in ApoE^−/−^ mice, while RSV treatment significantly reversed these phenomena (Figure 3F–K). These results collectively suggest that RSV effectively reduces atherosclerotic lesions by improving iron metabolism and inhibiting oxidative stress damage in the body.

### 3.3 Screening Target Genes of RSV‐AS‐Ferroptosis

**FIGURE 4:**
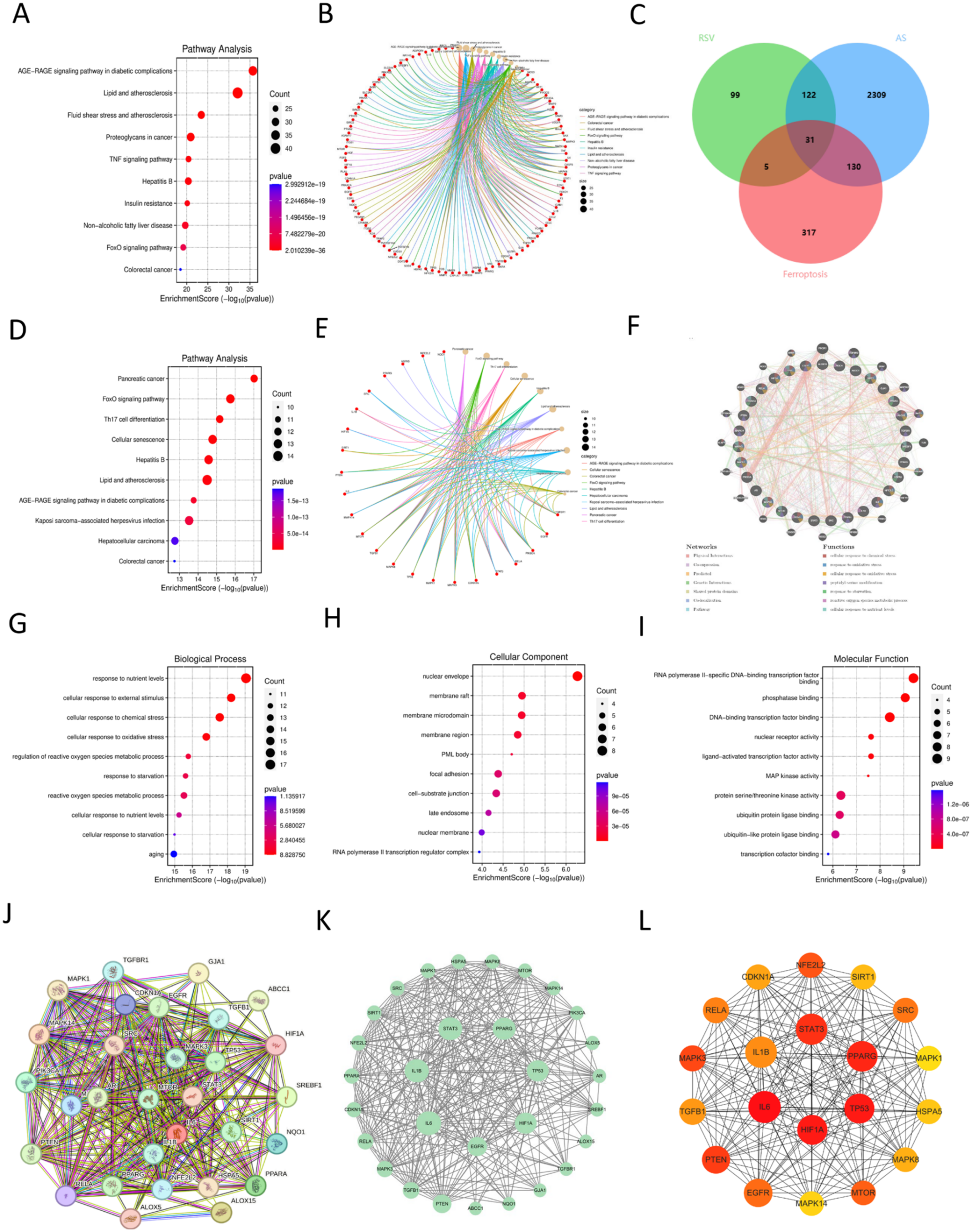
Predicted targets and mechanisms of RSV's anti‐AS effect by regulating ferroptosis. (A, B) KEGG enrichment pathway analysis of shared genes of RSV and AS; (C) Common genes in Venn diagram of RSV, AS, and ferroptosis genes; (D, E) KEGG enrichment pathway analysis of 31 related genes enrichment pathway analysis; (F) analysis of functional associations of targets using GeneMANIA; (G–I) GO enrichment map of 31 related genes, including BP, CC, and MF; (J) PPI network based on STRING database; (K) Cytoscape visualized PPI network of 31 genes; (L) Cytohubba plug‐in screened the Cytoscape visualized PPI network of the top 20 genes.

Through cross‐disease, GeneCards, OMIM, and GEO databases, we obtained 2592 genes associated with AS. The intersection of RSV and AS genes yielded 153 shared genes, which represent genes involved in RSV treatment of AS. To explore the potential mechanism of RSV action on AS, functional enrichment analysis of 153 shared genes was performed using KEGG. The results showed that the shared genes were mainly involved in related processes such as the regulation of the lipid biosynthesis process in atherosclerosis (Figure 4A,B). Ferroptosis, an iron‐dependent form of programmed cell death driven by abnormal lipid peroxidation, is closely related to the occurrence and development of AS. Therefore, we hypothesized that the underlying mechanism of RSV anti‐AS might be related to ferroptosis. We collected FRGs using FerrDb and identified 483 targets.

We identified 31 genes in the RSV and AS gene sets that were associated with ferroptosis, defined as shared FRGs (Figure 4C). To understand the biological functions and signaling pathways of common FRGs, GO, KEGG, and GeneMANIA functional analyses were performed to reveal the molecular functional relationships (Figure 4D–I). Next, we constructed PPI networks for 31 proteins using the STRING online tool (Figure 4J). Subsequently, the CytoNCA plugin was used to calculate the topological parameters and extract the core PPI network. The degree of the target is represented by the node size, from large to small (Figure 4K). At the same time, a pharmacological network representing the relationship between RSV‐FRGS‐AS was constructed, which provided a general understanding of the relationship between RSV, FRGs, and AS. We then calculated the top 20 hub genes using the Cytohubba plugin. These included RELA, IL6, SRC, MAPK14, PTEN, TP53, NFE2L2, SIRT1, EGFR, CDKN1A, MAPK8, HSPA5, HIF1A, TGFB1, STAT3, MAPK3, IL1B, MTOR, PPARG, and MAPK1 (Figure 4L).

### 3.4 Machine Learning Algorithms Identify Target Genes of RSV‐AS‐Ferroptosis

**FIGURE 5:**
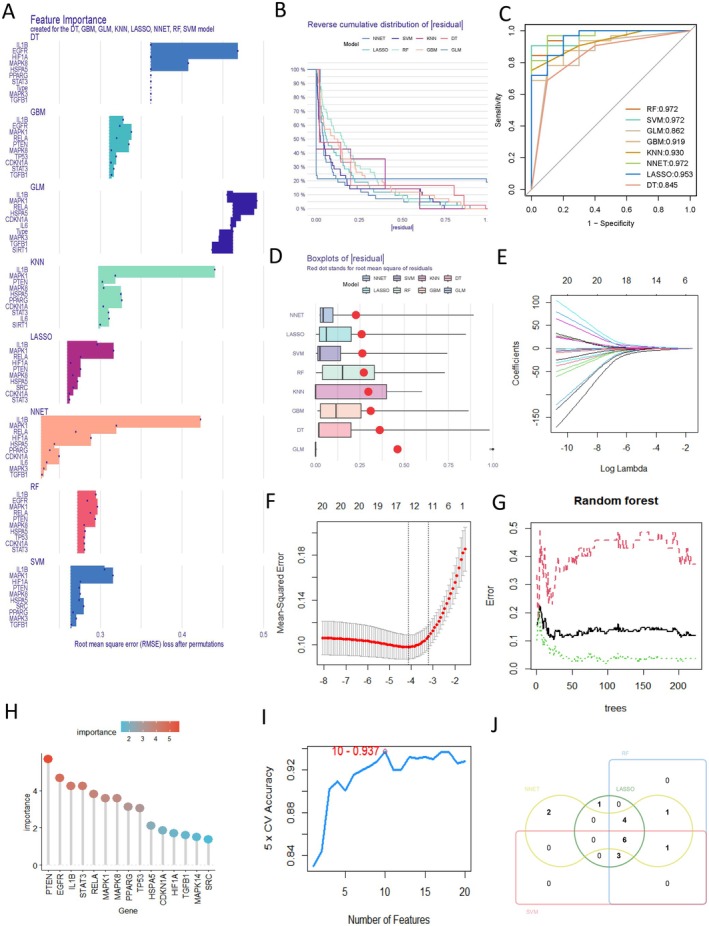
Machine learning screens six core targets. (A) Eight machine learning importance distribution plots of 20 hub FRGs calculated by Cytohubba, (B, D) residual plots of eight machine learning results, (C) ROC curves of eight machine learning methods. (E) LASSO regression of 20 hub FRGs calculated with Cytohubba, (F) cross‐validated LASSO regression parameter selection, (G, H) in RF analysis, random forest plots as well as significant gene visualization (score > 1.35). (I) SVM‐RFE algorithm was used to obtain FRGs with minimum error and highest accuracy, which were considered to be the most suitable candidate biomarkers. (J) Comparison between Venn diagram LASSO, RF, NNET, and SVM‐RFE algorithms showing shared/unique genes.

The study used eight machine learning algorithms (DT, GBM, GLM, KNN, LASSO, NNET, RF, and SVM) to model and analyze 20 hub FRGs calculated by Cytohubba (Figure 5A) and evaluate the reliability of the eight machine learning methods (Figure 5C). According to residual plots of eight machine learning results (Figure 5B,D), the top four scoring models were selected to evaluate 20 hub FRGs, and the best 14 biomarkers were obtained by the LASSO algorithm. These included RELA, SRC, MAPK14, PTEN, TP53, NFE2L2, EGFR, CDKN1A, MAPK8, HSPA5, HIF1A, TGFB1, IL1B, and MAPK1 (Figure 4E,F). At the same time, the RF algorithm identified 15 potential biomarkers (PTEN, EGFR, IL1B, STAT3, RELA, MAPK8, PPARG, TP53, HSPA5, CDKN1A, HIF1A, TGFB1, MAPK14, SRC, and MAPK1) based on their importance (Figure 5G,H). In addition, SVM‐RFE analysis showed that the model involving 10 genes (IL1B, RELA, HIF1A, STAT3, EGFR, TP53, MAPK14, SRC, PTEN, and MAPK1) had the highest modeling accuracy (Figure 5I). The NNET algorithm was used to select the top 14 core genes (TGFB1, MAPK3, IL6, PPARG, HSPA5, CDKN1A, RELA, HIF1A, IL1B, SRC, STAT3, PTEN, MAPK8 and MAPK1). Finally, four machine learning algorithms cross‐identified genes to obtain six candidate biomarkers with relatively obvious expression in AS, namely MAPK1, RELA, HIF1A, IL1B, SRC, and PTEN (Figure 5J).

### 3.5 Target Genes for Differential Gene Analysis

**FIGURE 6:**
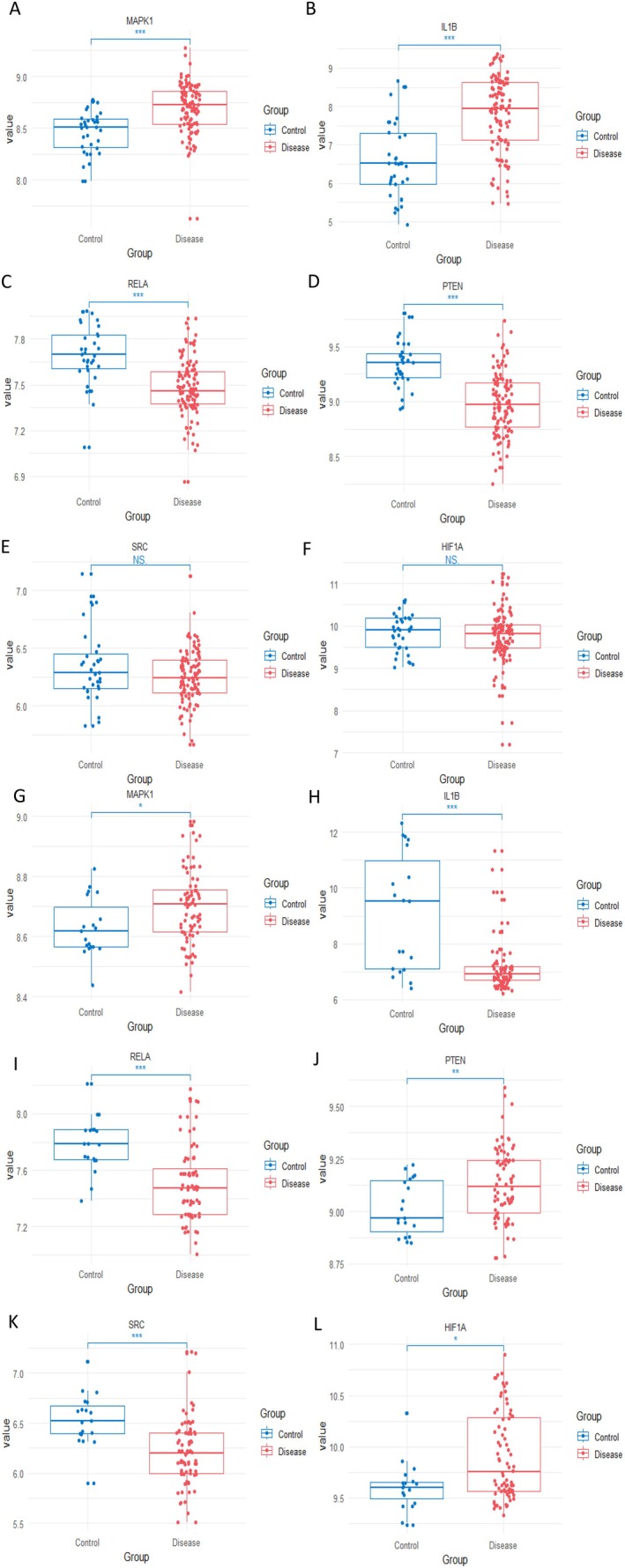
Expression of six core targets in differential gene analysis. (A–F) The expression of MAPK1, RELA, HIF1A, IL1B, SRC, and PTEN genes in the test set was analyzed by using R language DEGs. (G–L) DEGs analysis was used to verify the gene expression of MAPK1, RELA, HIF1A, IL1B, SRC, and PTEN in the AS group compared with the control group. Data are presented as mean ± SEM. *p < 0.05, **p < 0.01,***p < 0.001.

We analyzed six target gene DEGs by R language analysis, and test set AS tissues showed upregulation of MAPK1 and IL1B expression (Table 1), downregulation of RELA and PTEN expression, and no statistically significant differences in HIF1A, SRC compared with the control group (Figure 6A–F). In the validation set, compared with the control group, the expression of MAPK1, PTEN, and HIF1A was up‐regulated in the experimental group (Table 1), and the expression of IL1B, RELA, and SRC was downregulated (Figure 6G–L). The expression of MAPK1 gene was significantly upregulated in different samples compared with the control group.

**TABLE 1:** The information of TNBC‐related datasets.

Dataset	Platform	Total	AS	Normal	Note
GSE100927	GPL17077	104	69	35	Test dataset
GSE125771	GPL17586	40	40	0	Test dataset
GSE23746	GPL2700	95	76	19	Validation dataset

### 3.6 RSV Against AS by Reducing MAPK1 Expression

**FIGURE 7:**
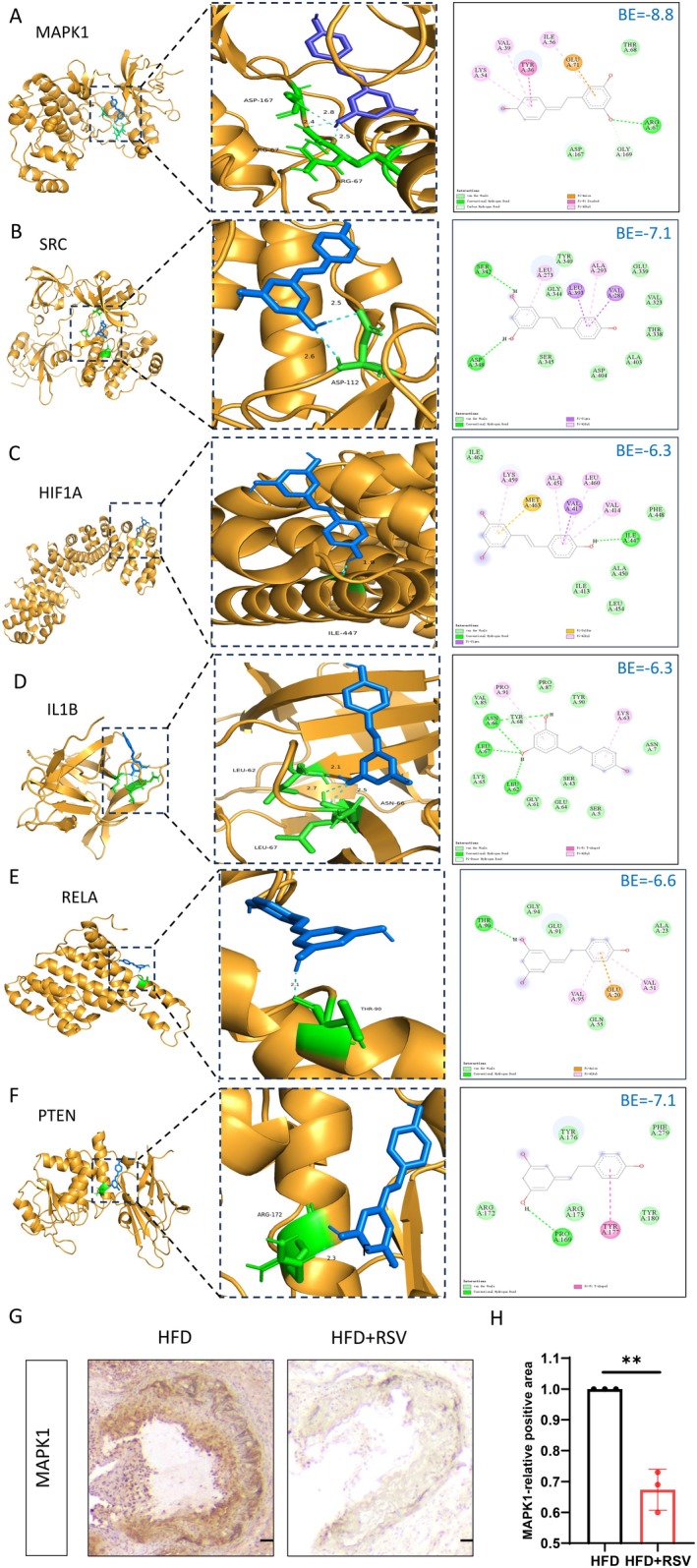
RSV against AS by reducing MAPK1 expression. Molecular docking of RSV with MAPK1 (A), SRC (B), HIF1A (C), IL1B (D), RELA (E), and PTEN (F). Immunohistochemical staining [scale bar = 250 μm] (G) and quantification (*n* = 3) (H) of MAPK1 protein expression level in mouse aortic roots. Data are presented as mean ± SEM. **p < 0.01.

To explore the potential of RSV active components to improve AS by modulating ferroptosis, we performed molecular docking analysis to investigate their interactions with potential ferroptosis therapeutic targets. Based on the network pharmacological bioinformatics and machine learning results, we identified six key biomarkers of RSV involved in the regulation of AS through ferroptosis for molecular docking. MAPK1 (−8.8 kcal/mol), IL1B (−6.3 kcal/mol), RELA (−6.6 kcal/mol), HIF1A (−6.3 kcal/mol), SRC (−7.1 kcal/mol), and PTEN (−7.1 kcal/mol), a score < −4.25 kcal/mol is generally considered to indicate a good affinity, and a docking score < −7 kcal/mol is considered to indicate a strong affinity (Figure 7A–F). We observed that RSV treatment significantly reduced MAPK1 expression in aortic roots of ApoE^−/−^ mice, with statistically significant results (Figure 7G,H). These results collectively suggest that RSV may reduces atherosclerotic lesions in the aorta and aortic root of ApoE^−/−^ male mice by reducing MAPK1 expression.

## 4 Discussion

Cardiovascular disease is a major global health threat, affecting more than 500 million people and causing about one third of deaths worldwide (Surma and Banach 2021). AS a chronic metabolic inflammatory disease involving the intima of large and medium‐sized arteries, AS is the main pathological basis of cardiovascular diseases such as coronary artery disease, cerebrovascular disease, and peripheral artery disease (Aprotosoaie et al. 2022). Its pathogenesis is complex, involving multiple pathophysiological processes such as lipid metabolism disorders, inflammatory cell infiltration, oxidative stress, and apoptosis. (Libby 2021) Although statins play an important role in lipid‐lowering therapy, their potential side effects and the residual risk of patients still exist. As the global burden of cardiovascular disease continues to increase, the development of safe and effective lipid‐lowering complementary therapies to prevent the complications of atherosclerosis has become a major medical problem that needs to be solved urgently (Omari and Alkhalil 2024).

As a traditional medical system with a long history, traditional Chinese medicine (TCM) has shown significant anti‐inflammatory effects in regulating physiological processes such as apoptosis, lipid metabolism, and oxidative stress, especially in preventing the formation of atherosclerotic plaques. In the field of natural medicine research, plant‐derived active ingredients have always been an important direction for the development of anti‐diabetes drugs. Among them, resveratrol (3,4,5‐trihydroxystilbene), as a typical polyphenolic phytoalexin, is widely found in grapes, berries, peanuts, and other plants, and has attracted much attention because of its excellent metabolic regulation function. This compound not only has a good safety profile but also has been widely used in the field of nutritional supplements due to its significant anti‐inflammatory and antioxidant properties. In recent years, with the deepening of pharmacological research, the potential value of resveratrol in the prevention and treatment of cardiovascular diseases has become increasingly prominent. A large number of studies have confirmed that this substance plays an important protective role in the prevention and alleviation of cardiovascular diseases and their complications through its antioxidant and anti‐inflammatory dual mechanisms (Beaudoin et al. 2014). Atherosclerosis is a chronic inflammatory disease. Resveratrol exerts a significant anti‐inflammatory effect by inhibiting key inflammatory signaling pathways such as NF‐κB and MAPK. At the same time, resveratrol also has strong antioxidant properties, which can reduce the formation and progression of atherosclerotic plaques by scavenging free radicals, reducing the generation of reactive oxygen species (ROS), and activating the endogenous antioxidant enzyme system. It has shown a significant protective effect in a variety of atherosclerotic cerebrovascular diseases such as coronary atherosclerotic heart disease, hyperlipidemia and diabetes (Su et al. 2022).

Studies have shown that the pathological process of AS is closely related to a variety of cell death mechanisms, including necrosis, apoptosis, autophagy, and the newly discovered ferroptosis. HFD could significantly induce ferroptosis in the aorta and aortic arch of mice, which is accompanied by obvious atherosclerotic lesions and a hyperlipidemia phenotype. As a newly discovered iron‐dependent programmed cell death, ferroptosis plays an important role in the occurrence and development of atherosclerosis by increasing the level of ROS and promoting lipid peroxidation (Li et al. 2024). Related studies have shown that resveratrol can inhibit ferroptosis and improve cardiac function through the Sirt1/p53 pathway (Zhang et al. 2023). So, does RSV effectively improve AS by inhibiting ferroptosis?

We obtained 1481 AS targets by collecting the OMIM and GeneCards databases and using the continuous median Relevance score. A total of 2092 differentially expressed genes were identified by DEG analysis from 144 samples of GEO database GSE100927 and GSE125771 datasets. By cross‐collection of DEGs identified by WGCNA and Limma, 1301 AS related genes were obtained. By crossing AS with RSV‐related genes, 153 RSV and AS core targets were obtained (Figure 2). Four hundred eighty‐three FRGs were collected by FerrDb, a database of ferroptosis related markers, regulators and management and identification, and 31 cross genes were selected from the RSV‐AS‐Ferroptosis, were analyzed by KEGG and GO pathway enrichment analysis. PPI was analyzed by STRING database Network findings were strongly associated with FOXO and other pathways and oxidative stress lipid production (Figure 4). We calculated the top 20 hub genes using the Cytohubba plugin, combined with eight machine learning algorithms to select four potential biomarkers (IL1B, RELA, HIF1A, SRC, PTEN, and MAPK1) (Figure 5) and performed molecular docking to verify their affinity. Among them, MAPK1 (−8.8 kcal/mol) binding capacity was better (Figure 7), compared with the control group, differential gene testing and validation were significantly increased (Figure 6).

Ferroptosis plays an important role in the treatment of AS, and MAPK1 enhances ferroptosis (Huang et al. 2021; Pan, Gan, et al. 2024). Studies have shown that RSV inhibits MAPK signaling pathway to alleviate ferroptosis (Chen et al. 2024). So, could RSV inhibit MAPK signaling pathway to alleviate atherosclerotic ferroptosis and improve AS? To investigate the effect of RSV on AS, ApoE^−/−^ mice were fed a HFD diet for 12 weeks to induce AS. Treatment with RSV significantly attenuated aortic and aortic arch atherosclerotic lesions (Figure 3C–E) and significantly reduced MAPK1 expression in the aortic root. Some studies have reported that the overload of intracellular Fe content increases the types of intracellular lipid reactive oxides and induces ferroptosis (Li et al. 2021). We found that RSV treatment reduced serum Fe in AS model mice (Figure 3F–K), inhibited SOD enzyme activity and its inhibition rate, reduced MDA expression, GSSG and GSSG/GSH expression, and improved the oxidative stress state of the body. In conclusion, RSV may play an anti‐atherosclerosis role by inhibiting ferroptosis to improve oxidative stress damage and reduce AS plaque formation through MAPK1.

Therefore, based on our study, the gap in the research of how RSV regulates ferroptosis against AS was filled to explore the potential molecular mechanism of RSV‐induced ferroptosis as a clue. Our study presents several novel findings. Firstly, we demonstrated that RSV might inhibit ferroptosis to exert anti‐atherosclerosis effect; In addition, RSV regulates ferroptosis mainly through six biomarkers, including IL1B, RELA, HIF1A, SRC, PTEN, and MAPK1, in AS treatment. Our study shows that RSV inhibits ferroptosis through MAPK1 to improve oxidative stress damage and reduce AS plaque formation, which plays an important role in the diagnosis and prevention of AS.

## 5 Conclusion

Our study suggests that RSV plays an anti‐AS role by inhibiting ferroptosis to improve oxidative stress damage and reduce AS plaque formation through MAPK1. This study combined RSV with AS and ferroptosis for the first time, comprehensively explored MAPK1 AS a new target for the treatment of AS and related mechanisms, and provided a theoretical basis for the application of RSV in AS.

## Author Contributions

**Yao Zhang:** conceptualization, methodology, software, writing – original draft preparation. **Jun Cheng:** carefully checked the pictures and tables in the manuscript, corrected and revised the content of the manuscript, and provided financial support. **Wu Jian:** writing – reviewing and editing.

## Ethics Statement

This study does not involve any human trials.

## Conflicts of Interest

The authors declare no conflicts of interest.

## Data Availability

The data that support the findings of this study are available from the corresponding authors upon reasonable request.
